# Development of a wearable global positioning system for place and health research

**DOI:** 10.1186/1476-072X-7-59

**Published:** 2008-11-25

**Authors:** Daniel Rainham, Daniel Krewski, Ian McDowell, Mike Sawada, Brian Liekens

**Affiliations:** 1 McLaughlin Centre for Population Health Risk Assessment, University of Ottawa, Ottawa, Ontario, Canada; 2 Department of Epidemiology and Community Medicine, University of Ottawa, Ontario, Canada; 3 Department of Geography, Laboratory for Applied Geomatics and GIS Science (LAGGISS), University of Ottawa, Ottawa, Canada; 4 Department of Civil Engineering, Dalhousie University, Halifax, Canada

## Abstract

**Background:**

An increasing number of studies suggest that characteristics of context, or the attributes of the places within which we live, work and socialize, are associated with variations in health-related behaviours and outcomes. The challenge for health research is to ensure that these places are accurately represented spatially, and to identify those aspects of context that are related to variations in health and amenable to modification. This study focuses on the design of a wearable global positioning system (GPS) data logger for the purpose of objectively measuring the temporal and spatial features of human activities. Person-specific GPS data provides a useful source of information to operationalize the concept of place.

**Results:**

We designed and tested a lightweight, wearable GPS receiver, capable of logging location information for up to 70 hours continuously before recharging. The device is accurate to within 7 m in typical urban environments and performs well across a range of static and dynamic conditions.

**Discussion:**

Rather than rely on static areal units as proxies for places, wearable GPS devices can be used to derive a more complete picture of the different places that influence an individual's wellbeing. The measures are objective and are less subject to biases associated with recall of location or misclassification of contextual attributes. This is important for two reasons. First, it brings a dynamic perspective to place and health research. The influence of place on health is dynamic in that certain places are more or less relevant to wellbeing as determined by the length of time in any location and by the frequency of activity in the location. Second, GPS data can be used to assess whether the characteristics of places at specific times are useful to explaining variations in health and wellbeing.

## Background

The notion of place in health research is both a spatial unit of analysis and a context that comprises the physical resources, exposures and social relations that may support or weaken health status. As a spatial unit, place is a space with boundaries commonly used for categorizing and discretizing predictors of health status. As context, places can be defined by the significance and meanings people attach to locations where health promoting or health suppressing activities occur. The idea of place as a determinant of health status has recently become a crucial focus of national population health initiatives [1,2]. An increasing number of empirical studies in medical geography and epidemiology have determined that characteristics of place are associated with variations in health-related behaviours and outcomes, even after individual-level attributes and behaviours are taken into account [3-5]. Although statistical associations between characteristics of place and health can be demonstrated, the underlying mechanisms responsible for these relationships remain more elusive. The significant challenges for place-based health research are to ensure that places are accurately represented spatially, and to identify those aspects of context that are related to variations in health and amenable to modification. This study focuses on the more practical issue of spatial bounding as a necessity to operationalize the concept of place. We introduce the development and testing of a wearable global positioning system (GPS) data logger for the purpose of objectively measuring the spatial extent of an individual's location over time. Examination of time-location data allows for inference on the types of activities associated with health status.

There are three principal methods of spatial bounding that dominate the previous literature on place and health research. Most studies make use of existing administrative boundaries, usually created *a priori *by national statistical or postal services. For example, analyses from the United States and Canada usually employ census tracts – small and relatively stable statistical divisions that vary in size by the density of settlement in urban areas [6,7]. Another approach defines places qualitatively according to boundaries and community assets as perceived and defined by their inhabitants [8-10]. More recent studies are defining place according to results derived from manual or automated zoning procedures [11,12]. Place boundaries can be manually determined by statistical design rules to assemble small geographical building blocks into larger regions so as to control population size, or another denominator of interest. Alternatively, basic spatial units can be grouped into larger ones automatically using automated zoning software.

Existing methods of spatial bounding are subject to several limitations. First, the majority of studies assume that the relationships between context and health operate within the confines of a single spatial unit, usually represented by an individual's residential census tract. This assumption may lead to the misclassification of context to variations in health status since it is unlikely that a person would spend all of their time in their residential census tract. Places that influence health are more likely to be spatially interdependent, linked by functional, cognitive and, possibly, sentimental relationships between what happens at one point in space and what happens elsewhere [13]. People live and function in various places that interconnect in complex ways, and to represent place as a single spatial area risks losing important exposure information. Second, researchers must ascertain the spatial scale appropriate for analysis, the level of aggregation characteristic to the data available, as well as the appropriate temporal frame within which to study causal relationships. Publicly-available datasets are usually static in space and time, and data is routinely collected without consideration of spatial or dynamic process [14]. Moreover data for health research are limited by lack of attention to spatiality, specifically how the spatial-temporal structuring of daily life defines how social action and relationships are represented [15]. Third, the scale of observation can influence inference [16,17]. This effect is called the "modifiable aerial unit problem" and, because changing the shape or size of the units on which data are mapped will change average values of the variables recorded, this can change the resulting correlations or statistical models generated from the data [18]. Spatial units such as postal codes or census tracts may also be changed over time, so altering statistical estimates [6,13,19-22]. Several solutions to the problem have been proposed, including statistical bounding [23], multi-scale and zone sensitivity analyses [24], and spatially-weighted regression techniques [25,26].

An alternative approach to delineating spatial boundaries makes use of time-location data. Recording changes in spatial location through time ultimately provides the most complete source of evidence on how place may influence health. All human activities have spatial and temporal dimensions: activities occur at particular places for limited durations [27,28]. By capturing simultaneously the locations and activities individuals through time it is possible to construct a series of space-time paths that represent objectively both spatial extent and the intensity of activity (as represented by time) in one or more places. Information about time, location and activity is usually acquired from interviews, personal observation (shadowing) or through time-diaries [29]. Other approaches using electronic sensors and loggers have been employed quite successfully in the context of transportation research and time-activity studies [30-33]. Recent efforts have improved on GPS tracking technologies for the purpose of measuring harmful exposures in human health research [34,35]. However, many of these studies have faced limitations in GPS accuracy, battery capacity and data logging memory thus preventing the collection of time-location data over extended periods of time (> 1 week) under a variety of environmental conditions. These limitations also make it difficult to capture individual time-location information in a variety of contexts. The present article describes the development of a new time-location measurement tool suitable for studies where information about location and time is used in predicting variation in human health outcomes.

### Global Positioning Systems

In 1995 the US Department of Defence (DOD) developed a satellite-based radio navigation system capable of determining within centimetres a position on the earth's surface. The system consists of 24 active and several "back-up" satellites, that orbit the earth providing all-weather navigation and surveying worldwide [36]. A summary of GPS capabilities is provided here, but for detailed information readers are referred to *Global Positioning System: Theory and Practice *by Hoffman-Wellenhof et al [37].

Global positioning systems (GPS) consist of three components: satellites in space, a ground control system, and the user's instrument. The space component consists of orbiting GPS satellites equipped with atomic clocks, and transmitting two radio frequencies modulated with two types of code: precise and standard. The precise code is reserved for U.S. military operations while the standard code can be used freely by any civilian in possession of a GPS receiver. The civilian code comprises a 50 bs^-1 ^radio signal transmitted at 1,575.42 MHz carrying three signals: a pseudo-random code, ephemeris data, and almanac data. Together these provide information on the satellites available for fixing a position, the current time and date, and the approximate constellation of the satellites at any time throughout the day. The ground control segment consists of five monitoring stations, three ground antennas, and a master control station. The monitoring stations passively track all satellites in view and accumulate ranging information. This information is processed at the master control station to determine satellite orbit geometry and to update the navigation message broadcast by each satellite. The user segment of the system consists of GPS receivers that calculate their own distance from each satellite based on the travel time of the pseudo-random sequences encoded into the radio signal. Given the geometric positions of the satellites (their ephemeris), four pseudo-ranges are sufficient to correct clock error and to compute the three dimensional position of the receiver with an average accuracy of approximately 10 m [38,39]. Most modern GPS receivers can track 12 or more satellites simultaneously, improving positional accuracy to within 5 m or less.

Natural Resources Canada, a federal government agency, manages a network of ground stations that transmit differential GPS (DGPS) corrections. These ground stations receive satellite information at a known ground location and estimate the difference between the information received by the satellite receiver and the actual location. Differential-enabled GPS receivers can receive the broadcast DGPS signals from the ground stations and make correction calculations, improving accuracy of position to within 3 m or less. Correction data are also available as public domain information from the Canada-wide differential GPS service, the International GPS Service and the Canadian Coast Guard. GPS signal correction can be performed at the time of measurement, using a DGPS receiver, or data may be post-processed if information on both the position of the DGPS receiver and ground station are collected. Improvements in location accuracy can also be achieved through the reception of signals from wide area augmentation systems (WAAS) and researchers should check to see if this service is available in their geographic region.

GPS signals are not immune to interference. The most severe form can occur from intentional signal degradation, also called "selective availability" by the United States National Space-Based Positioning, Navigation and Timing (PNT) Executive Committee [37]. Intentional, slowly changing random errors could be introduced into the pseudorandom code transmitted by each satellite resulting in substantial reductions in positional accuracy of 50 m or more in both the horizontal and vertical directions [40]. However, selective availability was removed from the system under executive order on May 1, 2000. The United States Department of Defence has since declared selective availability will no longer be used based on security concerns.

Several additional sources of interference may introduce errors that limit the usefulness of GPS in some spheres of human health and activity research. Variability in atmospheric conditions may affect the velocity of GPS signals. In the troposphere water vapour can slow radiofrequency signals resulting in overestimation of signal range. In the ionosphere different components of a signal can be advanced or delayed when interacting with charged gases. The sum of these atmospheric effects can result in errors of 30 to 60 m and vary depending on the angle of inclination of the satellites in view [41]. These effects are greater for satellite signals nearer the horizon where signals travel further through the atmosphere before reaching the GPS receiver. Of particular interest to human tracking studies are multi-path errors arising from the reflection of satellite signals from other surfaces, including buildings, vegetation, the ground or water. GPS signals are also blocked by materials such as concrete and steel thus eliminating reception within many institutional and commercial buildings. GPS reception is nonetheless relatively good in automobiles and public transportation vehicles such as buses and trains. GPS accuracy may also be compromised by poor satellite geometry, viewed from the user's location; precision is greatest when signals are received from satellites that are widely dispersed in azimuth and elevation. Thus, two satellites in the same location relative to the antenna provide similar information. The influence of satellite geometry can be quantified using dilution of precision (DOP) indices. Two DOP measures that are important for human tracking research include the positional and horizontal dilution of position (PDOP and HDOP). The first measure expresses uncertainty in overall position whereas the latter assesses uncertainty on the *x *and *y *axes. DOP measures generally range from 1 to 10 so that a location estimated with an HDOP of 2.6 has an uncertainty in the horizontal position that is approximately 2.6 times that of the receiver capability.

**Figure 1:**
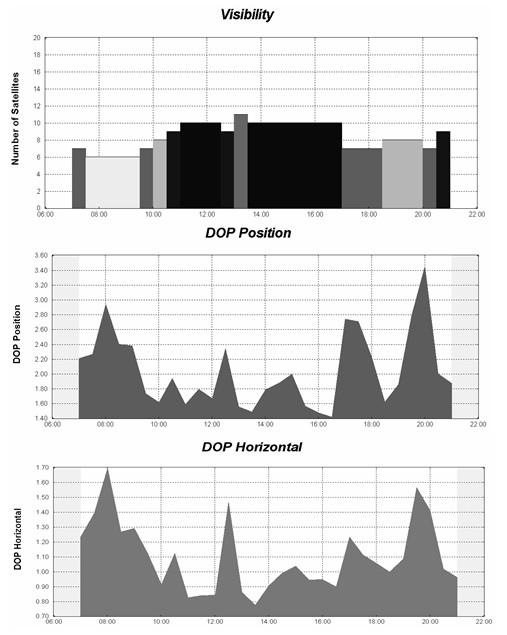
The number of satellites and dilution of position measurements (PDOP and HDOP) for September 25, 2007, at a central location in urban Halifax, Nova Scotia. DOP values of less than 2 are more desirable.

Researchers and GPS users interested in minimizing positional errors can undertake a mission planning exercise. GPS satellite orbits are known and predictable so that the number of available satellites and their geometric position can be computed for any location and any time. Mission planning software is freely available and almanac information can be downloaded electronically in multiple formats. Figure 1 is a typical report from mission planning software showing the number of satellites (visibility) and two DOP values (PDOP and HDOP)for 0700 to 2100 hours on September 25, 2007, at an urban location in Halifax, Nova Scotia. The opportunity for best reception is between 0900 and 1630 hours when seven or more satellites are available at most times within this window and PDOP values are less than 2.3.

### GPS Applications in Health Research

Advances in the miniaturization of GPS and related technologies have led to an assortment of applications: timing, surveying, logistics, traffic management and control, security, marketing, and navigation systems [42]. Of particular interest here is the development of GPS technology for health-related applications, specifically those concerned with navigation and tracking. Administrators of emergency 911 systems in many larger urban areas use geocoded address and GPS navigation systems to direct emergency response activities. The incorporation of assisted-GPS technology into cellular telephones allows emergency response teams to accurately assess the location of the distressed caller. The U.S. Federal Communications Commission (FCC) currently requires mobile phone service providers to locate emergency (E911) callers with an accuracy requirement of 100 m (67%) or 300 m (95%) for network-based solutions and 50 m (67%) or 150 m (95%) for handset-based solutions (usually GPS-enabled) [43]. Geocoded emergency 911 databases can be used to identify the location of an individual at a specific time; this information can be linked to contextual data to explore the role of place as a determinant of health emergencies. GPS in conjunction with other technologies has been used to support tuberculosis control programs in South Africa [44,45], and to identify high-risk areas for transmission of vector-borne and environmental diseases [46]. GPS is also used to investigate the positional accuracy of geocoding processing in epidemiological research [47,48]. Finally, microscale positioning systems that use three-dimensional imagery instead of satellite data are showing promise in surgical applications [49].

Although in its infancy, the use of GPS technology for human tracking presents an enormous opportunity for improving understanding of how the characteristics of places and environmental context influence human activity as well as health and well-being. Many technologies and techniques for human tracking have evolved from wildlife tracking research. GPS receivers have been used to track turtles [50], bears and other large mammals [51], farm and pastoral animals [52,53], and primates [54,55] with some success under a variety of landscape conditions. Very light GPS-enabled air pollution sensors have been fastened to homing pigeons in order to send real-time location-based pollutant information to an online database and mapping server (see ).

Innovations in GPS technology have cultivated interest in the development of portable and wearable GPS tracking devices for research on human activity. The majority of development in this area has been devoted to the commercialization of technologies for tracking criminals or persons under care of the courts. Titanium ankle bracelets with embedded GPS are routinely used for real-time tracking of prisoner transfers and for monitoring convicted offenders who are subject to restrictions on movement [56]. Several hardware vendors are retailing similar devices to monitor individuals with memory impediments (including Alzheimer's disease), children or individuals at risk of kidnapping, and family pets. Users can establish a "geofence" that generates an alert when a device moves beyond the limits of a predefined geographic boundary.

GPS technology has also been used in studies of physical activity and human exposures. Studies of exercise physiology and nutrition have used lightweight GPS receivers to assess physical activity as measured by the velocity of walking and running [38], to determine the mechanical power of walking [39,57], and to geographically contextualize accelerometry data, which indicates the locations where physical activity occurs [58]. Time diaries play an important role in epidemiological assessment of exposures to hazardous agents present in the environment. Several studies have employed commercially-available or custom designed wearable GPS data loggers to validate time-activity diaries [34,35] or to track individuals in studies of pesticide exposure [59]. More recently, GPS-enabled cell phones have been used to track adolescent travel patterns and activity information [60]. GPS technologies are now being linked with a variety of sensors to investigate relationships between environmental conditions and human physiology in time and space; these include sensors of environmental factors such as carbon monoxide concentrations or air temperature, and health-related factors such as heart rate [61,62].

The purpose of this study is to develop and pilot test a customized wearable GPS data logger suitable for tracking human subjects over lengthy periods of time. The ability to track people over extended periods of time facilitates the development of individualized spatial units for place and health analyses. In more urbanized areas the application of GPS technology to accurately measure location over time requires evaluative pilot testing for reliability and validity to ensure feasibility of the technology under actual conditions. Here we propose a general framework of dynamic and static tests for evaluating and testing human tracking devices based on GPS technology. A novel contribution of this work is the testing of a wearable GPS across multiple modalities of dynamic measurement among a variety of urban contexts. Knowledge of time-location patterns plays a critical role in understanding how people interact with, and use, space, and reveals the 'places' relevant to the study of variations in health outcomes.

## Methods

### Development of a Wearable GPS Data Logger

Technological features relevant to the development of wearable GPS for exposure assessment research have been discussed elsewhere [35,59]. The following list incorporates features from previous work, and introduces several additional physical and performance-based features judged to be critical for human tracking studies: a) size and weight (relatively light and unobtrusive, <0.5 kg), b) logging capability (configurable logging frequency and adequate data storage), c) run-time (minimum 2 d battery capacity at frequent sampling intervals, quick recharge), d) passive (simple to operate and requiring little or no interaction during logging), e) durable (resistant to vibration, minor impacts and water resistant), f) fast time-to-first-fix (obtain fix quickly after signal loss), g) accurate (2–5 m resolution and precise among a variety of built and natural environments).

**Figure 2:**
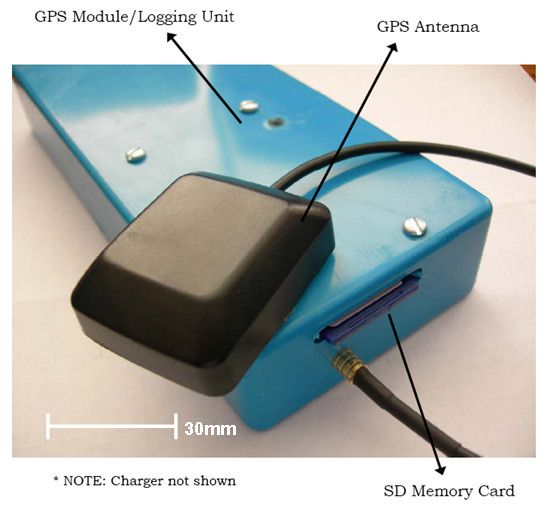
The HeraLogger, a wearable GPS data logger for health and place research.

After constructing several prototypes we developed a wearable GPS data logger instrument called the HeraLogger. The HeraLogger comprises a PVC case (165 mm × 71 mm × 25 mm) containing the GPS module, data logger and battery pack, and an external magnetic patch antenna (Figure 2). The instrument weighs approximately 170 g and easily fits into a jacket pocket or small bag. The antenna has a 2 m cable and can be positioned appropriately to maximize visibility of the sky and satellite signal reception. The GPS module can be configured to output position information at sampling rates up to a maximum of four times per second; data are logged to a removable SD card with capacity up to 1 GB. The instrument can accommodate multiple battery capacities, ranging from 2.4 Ah to 10.4 Ah; this range corresponds to 16 h to 71 h of runtime, or 57 600 to 248 400 data points using a one per second sampling rate. An on/off switch initiates the logging of geographic position and the instrument can be left on while recharging the batteries. The GPS module is a 16-channel receiver with a rated time-to-first-fix of less than 34 s for a cold start, less than 3.5 s for a hot start, and is accurate to within 2 to 5 m of its actual position [63]. Software was developed to read, parse and write satellite data to a text file suitable for import into a geographic information system or statistical software package. The cost of each instrument is approximately $450 not including labour costs associated with assembly. Four GPS instruments were assembled for further testing.

### Static Tests

**Figure 3:**
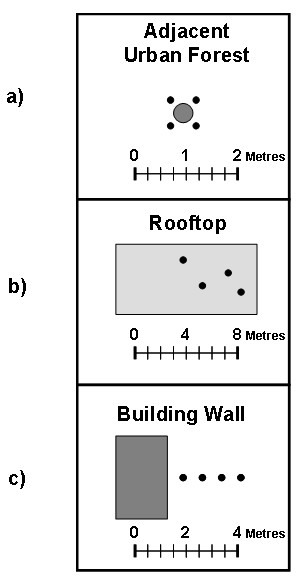
Configuration of GPS instruments for static testing: a) positioned adjacent to a survey monument; b) various rooftop locations at least 1 m apart; c) perpendicular to building wall at 1 m increments.

Static testing of the Heraloggers was conducted during the summer of 2007 at Dalhousie University, Point Pleasant Park and at the Art Gallery of Nova Scotia in Halifax, Nova Scotia. Three static tests were performed to assess instrument accuracy and precision under field conditions commonly experienced by human subjects. These conditions included the edge of an urban forested park (some obstruction from trees), an open rooftop with no obstructions, and near a building wall to simulate urban canyon conditions (Figure 3). GPS performance improves as the percentage of open sky increases [36].

Precision was estimated for all three sites; accuracy was assessed only at the park location using a known geodetic location, in this case a municipal survey monument maintained by the Province of Nova Scotia (Northing: 4940643.46, Easting: 454981.21, NAD83, UTM Zone 20 N). The four instruments were assessed simultaneously to control for the effects of weather (atmospheric interference) and variations in position estimation resulting from dilution of position (DOP). DOP refers to the geometric strength of satellite configuration on GPS accuracy [37]. None of the logged synchronous data were filtered for GPS signal quality so as to simulate actual field conditions. Sampling periods were not selected for optimal signal reception.

#### Urban Park Static Testing

The antenna from each GPS instrument was placed as close to the geodetic point as possible. The potential for inter-instrument interference was negligible given the use of passive antennas on each GPS device. Data were collected at 5 s intervals for a 1 h period resulting in a total of 720 data points per instrument for analysis. The average of all logged coordinates was compared with the known geodetic point to obtain an estimate of the accuracy of each instrument and to evaluate the total mean error from all four instruments. An additional test was performed to evaluate instrument precision. Data were collected at 5 s intervals for a 2 h period in a location in close proximity to the geodetic reference position. Precision was measured in terms of the standard deviation of the measured coordinates for each instrument.

#### Rooftop Static Testing

To evaluate the static performance of the GPS instruments under open sky conditions, four instruments were placed in watertight containers and positioned in a random formation (with a minimum distance between instruments of 1 m) on the rooftop of a building (Northing: 4943129.5, Easting: 453208.7, NAD83, UTM Zone 20 N) at Dalhousie University. An effort was made to select a rooftop of sufficient elevation to prevent interference of satellite signals (multipath errors) from adjacent structures. GPS data were collected over a 24 h period.

#### Building Wall Static Testing

To assess the impact of multipath errors arising from the influence of tall buildings characteristic of dense urban development (also known as the urban canyon effect), GPS instruments were placed at 1 m intervals in a straight line perpendicular to the outside wall of an eight story building (Northing: 4944229.0, Easting: 454802.6, NAD83, UTM Zone 20 N) in downtown Halifax. We hypothesize signal reception should worsen under 'urban canyon' conditions where the potential for multipath errors due to building interference increases. Data were again collected at 5 s intervals for a 1 h period.

#### Static Testing Analysis

Accuracy and precision based on the static tests were evaluated according to guidelines established by the Institute of Navigation and U.S. Department of Defence GPS specification documents [64,65]. Since the tracking of human movement focuses on the location of an individual at a point in time, only the horizontal accuracy and precision of the GPS instruments were calculated. All logged coordinates were converted from WGS-1984 geographic coordinates to UTM Cartesian coordinates (UTM, NAD83, Zone 20 N). The current version of WGS-1984 and the North American Datum of 1983 are equivalent; however, we note that the native instrumentation of the GPS system determines coordinates within the WGS-1984 reference system and thus, and transformations should be done in post-processing. The difference in horizontal accuracy and precision was calculated using equation 1:

(1) $$\Delta H={\left(\Delta e^{2}+\Delta n^{2}\right)}^{\frac{1}{2}},$$

where Δ*e *is the change in longitude [easting], and Δ*n *is the change in latitude [northing]. In both directions the change is dependent on a reference location. GPS instrument accuracy measures were derived from the forested park static test with the average of each instrument's logged coordinates compared to a known geodetic point. Precision measures were calculated using average of all logged points for each GPS instrument as the reference location so that coordinate point was compared to the mean of all logged coordinates. We assumed a Gaussian error distribution for measurements of latitude and longitude, which has been shown to be reasonably representative of coordinate measurements based on stand-alone GPS receivers [66]. The calculated difference values [Δ*H *] for each GPS instrument were ranked in order to apply the most common methods of comparing GPS accuracy, based on the circular error probable [CEP], horizontal accuracy distributions, and standard deviations in the x and y directions for each instrument [67,68]. CEP is the radius of a circle, centred at the antenna position, containing 50 percent of the points around the average value of all measurements [69]. Horizontal accuracy distributions were calculated as the radii of two circles, centred at the antenna position, containing 95 and 98 percent of all GPS points logged. S-plus statistical software was used for all calculations (S-Plus for Windows, Seattle, WA).

### Dynamic Tests

Positional data from each of the four GPS instruments were collected for four transportation modes: walking, cycling, automobile, and transit bus. Walking and cycling data were collected along a route approximately 5 km in length. The average time to complete the route was 66 min for walking and 24 min by bicycle. Test participants were asked to walk in the middle of the sidewalk, unless passing another pedestrian, and while cycling to maintain a consistent distance away from the curb unless changing lanes or turning. The automobile test route was approximately 40 min in length; drivers were instructed to abide by posted speed limits and to select the lane closest to the curb on roads with more than two lanes. Transit bus data were collected along an urban downtown bus route with a total loop time of approximately 80 min. Riders were not provided with any specific instructions regarding seating placement in order to avoid special efforts to improve signal reception by selecting a window seat.

#### Dynamic Testing Analysis

**Figure 4:**
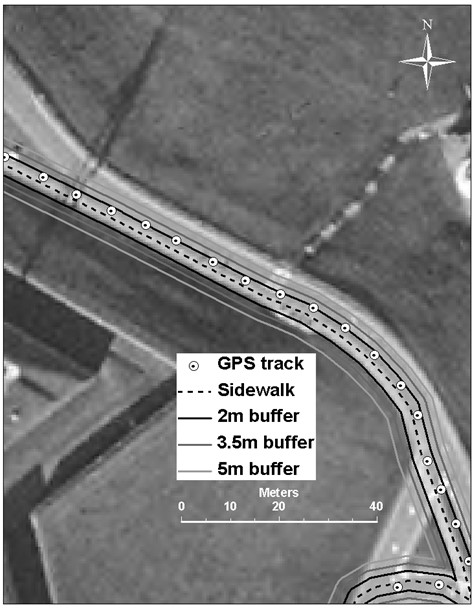
**A sample of points logged by the GPS instrument during a 45 minute walking test in open sky or optimal reception conditions. The true path (dotted line) is the sidewalk.** Most points fall within 2 m of the true path.

All positional data were converted into Cartesian coordinates and imported into a geographic information system for further analysis. Digitally orthorectified aerial photographs [70] were used to determine the true path coordinates by creating polyline themes for each transport mode route. True paths were in the middle of the sidewalk for the pedestrian data, within 1 m of the curb for cycling data, and in the middle of the lane for automobile data (except when lanes were crossed for turning). A similar process was used for the bus route, using spatially-referenced route data supplied by Halifax Regional Municipality served as a guide. Variations in the widths of the sidewalks and roads were accounted for when determining the true path polylines. GPS data from each transportation scenario were categorized according to three types of built environment: mixed density, open sky, and urban canyon. The route of the transit bus did not allow for the collection of GPS data under open sky conditions. Buffers of 2, 3.5, and 5 m were created on either side of the true paths and coordinates were analyzed to determine the percentage of points recorded inside each buffer distance and in each built environment type (Figure 4). The numbers of satellites used to determine location and dilution of position measures (HDOP and PDOP) were recorded directly from GPS output.

## Results

### Static Tests

Table 1 shows the number of points logged by each HeraLogger to determine the accuracy of the instruments. The average duration of the test was 68 min. The loggers received information from an average of 10.3 satellites during the course of this evaluation resulting in accurate position estimates and nominal PDOP and HDOP values (i.e. below 4.0 and 2.0 respectively). Results revealed horizontal position accuracies of 2.65 ± 0.25 m for 50% of logged values, 7.83 ± 1.17 m for 98% of logged values, and an average distance of 2.82 ± 0.40 m away from the known reference location.

**Table 1:** Horizontal accuracy comparison of four GPS instruments from the Forested Park test

						Accuracy Description		
Logger	N	Satellites^a^	HDOP^a^	PDOP^a^	CEP^a^	H95^a^	H98^a^	Distance^b^
6	825	10.3	1.19	1.90	2.60	5.00	8.00	3.33
14	819	9.9	1.44	2.34	3.00	6.00	7.20	3.00
15	814	10.4	1.37	2.03	2.60	6.90	9.40	2.49
23	811	10.4	1.17	2.06	2.40	5.60	6.70	2.53

Mean ± SD^c^	817	10.3 ± 0.23	1.29 ± 0.13	2.08 ± 0.18	2.65 ± 0.25	5.88 ± 0.79	7.83 ± 1.17	2.82 ± 0.40

^a ^HDOP = horizontal dilution of position; PDOP = positional dilution of position; CEP = circular error probable (50%) in metres; H95 and H98 = horizontal accuracy distribution at the 95 and 98 percent levels in metres. All are averaged values.

^b ^Distance in metres between geodetic reference point and average of all recorded GPS data.

^c ^Standard deviation

Table 2 gives details of the precision of the GPS instrument under three built environment scenarios. Precision was best in the park setting. The average duration of the logs was 115 min, and the mean number of satellites obtained, as well as HDOP and PDOP values, were similar to those data from the accuracy tests. Half of the values lie within 2.00 ± 0.35 m and 98% lie within 5.45 ± 1.06 m. Logged data from the rooftop location are to some extent less precise than those from the urban park location. Logging took place over a 24 h period and, on average, one less satellite was used to provide a location solution. The mean CEP value is 2.35 ± 0.11 m and 98% of the location data fall within 6.18 ± 0.29 m. Position precision estimates worsened under urban canyon conditions where multipath effects are expected to be more of an issue. Data were logged on average for 63 min; three fewer satellites were available to derive location solutions, in comparison with the urban park test. PDOP values almost doubled compared to other locations. The radius of the circle required to capture 50% of the position data was roughly nine times larger than for data from the urban park. A circle with an average of radius of 53.14 ± 25.06 m captured up to 98% of the GPS data.

**Table 2:** Horizontal precision estimates of four GPS instruments in three built environment types

*Urban Park*							
					Accuracy Description		
GPS ID	N	Satellites^a^	HDOP^a^	PDOP^a^	CEP^a^	H95^a^	H98^a^
6	1371	10.3	1.00	1.87	1.40	3.30	4.00
14	1398	10.3	1.00	1.83	2.20	4.40	4.90
15	1380	10.4	1.03	1.89	2.30	4.90	6.20
23	1374	10.3	1.06	1.93	2.10	5.00	6.70

Mean ± SD^c^	1380	10.3 ± 0.0	1.02 ± 0.02	1.88 ± 0.04	2.00 ± 0.35	4.40 ± 0.67	5.45 ± 1.06

*Rooftop*							
6	17284	9.9	1.07	1.89	2.50	5.10	5.90
14	17947	9.4	1.10	1.85	2.20	5.00	5.90
15	17946	9.2	1.09	1.87	2.30	5.20	6.30
23	17944	9.3	1.06	1.82	2.40	5.60	6.60

Mean ± SD^c^	17780	9.4 ± 0.3	1.08 ± 0.02	1.86 ± 0.03	2.35 ± 0.11	5.23 ± 0.23	6.18 ± 0.29

*Urban Canyon – Wall Test*							
1	762	7.2	1.43	3.53	11.40	48.00	69.10
2	770	7.3	1.40	2.81	20.10	24.60	24.60
6	755	7.7	1.49	2.95	9.50	57.90	78.00
15	774	7.5	1.43	2.96	13.40	34.10	39.10

Mean ± SD^b^	764	7.3 ± 0.2	1.49 ± 0.04	3.16 ± 0.32	14.04 ± 4.62	43.46 ± 14.73	53.14 ± 25.06

^a ^HDOP = horizontal dilution of position; PDOP = positional dilution of position; CEP = circular error probable (50%) in metres; H95 and H98 = horizontal accuracy distribution at the 95 and 98 percent levels in metres. All are averaged values.

^b ^SD = Standard deviation

### Dynamic Tests

Table 3 shows the performance of the GPS instruments using different forms of transportation in the three built environments. Positional accuracy under open sky conditions was best when cycling and walking (72% to 99.1% of all positions within 5 m). No data are available for transit bus tests in open sky conditions. In mixed density areas, determinations of position were more accurate from automobile logs (89% within 5 m), followed by cycling (81% within 5 m), walking (74.5% within 5 m) and then transit bus (65% within 5 m). In urban canyon areas where GPS receivers are most challenged, the greatest positional accuracy was attained from automobile (82.6% within 5 m) and transit bus modes (60.2% within 5 m), followed by walking and then cycling (57% and 53.7% within 5 m, respectively).

**Table 3:** Resolution estimates of four GPS instruments derived from four transportation modes in three built environment types

*Walking Tests*																
					Fraction of points within each buffer (%)											
					Mixed Density				Open Sky				Urban Canyon			
					
GPS ID	N	Sats HDOP		PDOP	N	± 5 m	± 3.5 m	± 2 m	N	± 5 m	± 3.5 m	± 2 m	N	± 5 m	± 3.5 m	± 2 m
6	824	8.9	1.47	3.08	419	91.4	78.5	53.0	189	100.0	98.9	85.7	216	71.3	64.8	52.8
14	821	8.6	1.96	2.70	412	52.4	40.8	27.4	203	95.1	82.8	53.2	206	43.2	35.9	20.9
15	702	9.9	1.43	2.87	355	84.5	73.0	53.8	167	98.8	97.6	87.4	180	62.2	56.1	43.3
23	826	9.1	1.51	3.02	418	87.1	80.1	61.5	189	98.9	94.2	81.0	219	61.2	56.6	41.1

Mean	800	9.2	1.68	2.88	403	74.5	63.2	44.5	190	96.9	90.8	72.0	206	57.0	49.2	35.0

																
*Bicycle Tests*																
6	294	8.7	1.58	2.61	140	90.0	74.3	47.9	81	100.0	97.5	76.5	73	50.7	34.2	16.4
14	297	9.1	1.52	2.49	141	73.8	58.2	38.3	80	98.8	97.5	87.5	76	53.9	43.4	30.3
15	296	8.2	1.61	2.66	135	78.5	65.9	41.5	81	98.8	91.4	64.2	76	60.5	42.1	25.0
23	293	8.4	1.55	2.53	139	82.0	61.2	38.1	82	98.8	90.2	70.7	72	50.0	41.6	20.8

Mean	295	8.6	1.57	2.57	138	81.0	64.9	41.4	81	99.1	94.1	74.7	74	53.7	40.3	23.1

																
*Automobile Tests*																
6	454	10.1	1.28	2.69	296	94.3	87.5	69.6	63	92.1	76.2	49.2	95	83.2	69.5	60.0
14	454	9.9	1.27	2.65	296	94.9	83.1	63.9	63	92.1	82.5	55.6	95	84.2	72.6	53.7
15	452	10.1	1.25	2.56	294	78.6	70.1	51.7	63	92.1	82.5	61.9	95	77.9	75.8	58.9
23	454	10.1	1.25	2.59	296	88.2	80.1	56.4	63	92.1	81.0	57.1	95	85.3	74.7	54.7

Mean	454	10.0	1.26	2.62	295	89.0	80.2	60.4	63	92.1	80.5	55.9	95	82.6	73.1	56.8

																
*Transit Bus Tests*																
6	1076	9.7	1.45	2.46	827	72.8	58.4	37.2					249	73.1	57.0	36.1
14	885	8.8	1.52	2.57	618	60.0	43.9	23.3					267	64.4	50.2	27.3
15	767	7.5	1.31	2.16	552	69.4	55.1	36.2					215	61.9	47.4	31.2
23	929	7.2	1.34	2.24	725	70.5	55.3	37.2					204	62.3	44.6	27.0

Mean	918	8.3	1.41	2.37	680	65.0	50.3	31.6					237	60.2	45.8	28.1

Satellite reception was best for automobile travel (10 satellites) followed by walking, cycling and then transit bus transportation (8.3 satellites); however, this difference in reception did not always translate into reduced horizontal and positional dilution of position values. Figure 4 shows a close-up perspective of a pedestrian path under open sky conditions. The dotted line shows the true path (sidewalk) bounded by 2 m, 3.5 m, and 5 m buffers, as well as the actual GPS locations logged at 5 s intervals. As expected, signal accuracy for walking and cycling deteriorated as the potential for the built environment to interfere with satellite reception increased. Differences in signal reception are less apparent under varying built environments for automobile trips.

**Figure 5:**
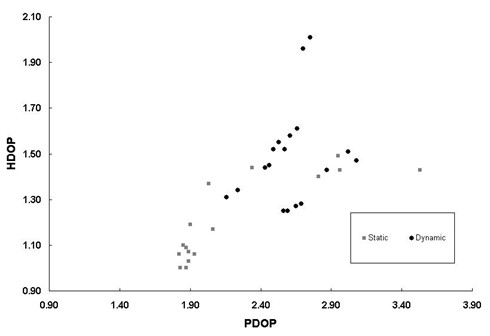
The relationship between HDOP and PDOP in static and dynamic accuracy testing conditions. Dilution of position increases under dynamic conditions.

Figure 5 shows the relationship between horizontal (HDOP) and positional dilution of position (PDOP) under static and dynamic GPS instrument testing conditions. Static HDOP varies in a log-linear fashion with PDOP, so that reductions in horizontal accuracy diminish at a value of approximately PDOP = 2.3; this implicates vertical dilution of position as the reason for decreased PDOP in static conditions. PDOP varied less with HDOP under dynamic conditions with no clear association between dilution of position values. HDOP values were greater under dynamic as compared to static conditions.

## Discussion

Finding an approach to accurately define living spaces is a problem inherent to place and health research. Because of the way existing data are presented, researchers are usually forced to adopt existing administrative boundaries; if these are inadequate they may have to develop alternative strategies to relate characteristics of place to explanations of health variations. Wearable global positioning systems (GPS) can accurately describe where individuals spend their time thus providing a more detailed assessment of places relevant to health. GPS data are also preferred to the use of time diaries as they minimize recall bias and may also reduce problems of compliance [34].

The objectives of this research were to develop and test a wearable GPS instrument for health and place research, as well as to establish a general framework of dynamic and static tests for evaluating and testing human tracking devices based on GPS technology. Specifically, we developed a passive wearable GPS receiver data logger that provided consistent time-location recording capabilities under a variety of static and dynamic conditions in an urban environment. Unlike devices used in previous research, the design characteristics of our GPS instrument allow for extended high resolution positioning under typical urban conditions and required little input or maintenance. The instrument can sample at 1 s intervals for approximately 70 h continuously before recharge, and there is no danger of approaching data storage limitations. Comparison with devices used in other studies is difficult due to the range of parameters selected by researchers, as well as a determination of what constitutes a "wearable" device. However, a similar device designed for assessing human exposures was able to record 25 h of data at 5 s intervals, and had a maximum logging capacity of 30 h [59].

Position accuracy and instrument precision under static and dynamic conditions in a variety of environments is critical for time-location analysis. There are a number of factors that influence GPS instrument position accuracy, most of which are unavoidable or beyond the control of the researcher; however, the influence of these factors is usually measurable. For example, researchers can investigate satellite constellation geometry to choose sampling times when dilution of position is diminished. Errors may also arise from atmospheric interference and instrument quality. Due to cost restrictions most survey-grade GPS (highly accurate) are not amenable to human tracking studies at this time.

The average accuracy of our GPS instrument is 2.8 m (± 0.4 m) when not in motion. This is a respectable degree of accuracy when compared to a range of 1.7 m to 10 m reported in similar studies [35,53,59,71], and is likely to be acceptable for most place and health studies. Instrument precision did not vary much between open and mixed density urban development conditions (98% of values lying within 5.5 m to 6.2 m of the true location). However, accuracy fell sharply in urban canyon settings (98% of values within 53.1 m of the true position). These values, which are similar to those obtained in similar evaluations [72], would inflate in larger cities with taller buildings and denser development.

GPS instrument accuracy under dynamic conditions is particularly relevant for place and health research, as well as for exposure assessment studies. People rarely remain in one location for extended periods of time (with the exception of sleeping), and movement is likely occur among a variety of locations and encompass multiple transportation modes. We evaluated the accuracy of a wearable GPS instrument in three types of urban environment across four transportation modalities. As expected, instrument accuracy is greatest when the potential for interference is least, regardless of transportation mode. However, the impact of the environment was less pronounced for automobile and bus transit modes, with the latter mode having relatively poorer absolute accuracy, regardless of location in an urban area. We found that under dynamic conditions, positional accuracy tends to improve as the distance from potential interference increases. Automobiles and buses operate in roadways which are further from buildings and other objects than cyclists or pedestrians. In a study involving children from the Seattle area, two wearable GPS instruments were tested for position accuracy after a 4 km walk in the city. The researchers reported 96 percent of locations within 5 m and 78.8% within 2 m [59] compared to 76.2% and 50.5% from our tests. However, direct comparison is difficult since the instruments used in the Seattle study were switched on in advance of data collection (this ensures a good initial location fix), and the data were post-processed to correct for errors using differential signal data. No information about the potential for physical interference arising from urban structures and features was available in that study.

The wearable GPS instrument described here is sufficiently accurate to locate and correctly classify a variety of human activities. Ideally, position data would be obtained for all activities regardless of environment. However buildings constructed of impenetrable materials limit GPS tracking. Although we do not report on measurements undertaken indoors, preliminary data from an unpublished pilot study of 53 individuals suggests that indoor tracking is possible under some conditions. The inconsistency of reception indoors, and to some extent outdoors, is explained by building materials, proximity to windows or sky, distance from building walls, and potential interference from other electronic devices [73]. Specifically, indoor environments reduce satellite availability, accuracy (due to high noise and degraded geometry), positioning continuity, and reliability. The availability of high-sensitivity GPS (HSGPS) and assisted GPS (AGPS) will help to improve indoor positioning performance [74]; however, even under ideal indoor conditions, signal accuracy is limited to within 10 m for residential and 70 m for commercial buildings [43].

Clearly GPS tells us very little about the context of place or the places where activities occur. While the data may indicate a visit to a local pub, we have no qualitative information on whether the visit was a pleasant experience or not. Ultimately combining GPS position data with questionnaires, interviews or other forms of data collection would enhance our understanding of place and how it influences health and well-being.

A wearable GPS instrument such as the HeraLogger could be used in other health research settings, and may be able to provide novel insights into temporal and spatial processes underlying place and health associations. We have conducted a pilot project where GPS instruments were worn with passive air particle samplers to improve air pollution exposure assessments. Collecting GPS position information over a 7 d period could provide a crude but objective estimation of an individual's spatial footprint. In future work we intend to compare this footprint to boundaries traditionally used in place and health research to assess bias arising from misclassification of place. Also of interest is the development of measures created from GPS information relevant to processes associated with variations in health. For example, social capital researchers may find GPS data useful for investigating places where socialization does and does not occur outside of the home and work environments. What is apparent from the data we have collected to date is that GPS data and time-location analysis will enable a more objective characterization of human activity patterns, so that we may better understand the spatial and temporal processes underlying the determinants of population health.

## Conclusion

Wearable GPS instruments can monitor human activities. Spatial accuracy is adequate to locate individuals within distinct subenvironments and, with knowledge of location, it is possible to make some assumptions about activity. Rather than rely on static areal units as proxies for places, wearable GPS devices can be used to derive a more complete picture of the different places that influence an individual's well-being. The measures are objective and are less subject to biases associated with recall of location. The resulting data can be visualized using maps delineating the spatial and temporal boundaries traversed by individuals. This is important for two reasons. First, it brings a dynamic perspective to place and health research. The influence of place on health is dynamic in that certain places are more or less relevant to wellbeing determined by the length of time in any location and by the frequency of activity in the location. Second, data can be grouped by traditional health determinants to see if there are any consistent spatio-temporal patterns among groups with similar characteristics, or whether there are characteristics of places in time that comprise or can explain variation in health and wellbeing as distinct from social and economic health determinants. Overall the use of wearable GPS-enabled technologies represents a logical next step in the assessment of the association between place and health.

## Competing interests

The authors declare that they have no competing interests.

## Authors' contributions

DR conceived of the study, participated in the development and testing of the wearable GPS data logger, performed any analyses and drafted the manuscript. DK and IM helped to draft and revise the manuscript. MS participated in design of the wearable GPS data logger and revised the manuscript. BL led the development of the wearable GPS data logger. All authors read and approved the final manuscript.
